# Exploring Household Food Dynamics During the COVID-19 Pandemic in Morocco

**DOI:** 10.3389/fnut.2021.724803

**Published:** 2021-09-27

**Authors:** Hamid El Bilali, Tarek Ben Hassen, Chedli Baya Chatti, Aziz Abouabdillah, Si Bennasseur Alaoui

**Affiliations:** ^1^International Centre for Advanced Mediterranean Agronomic Studies (CIHEAM-Bari), Valenzano, Italy; ^2^Department of International Affairs, College of Arts and Sciences, Qatar University, Doha, Qatar; ^3^Department of Social Sciences, College of Arts and Sciences, Qatar University, Doha, Qatar; ^4^Department of Agronomy and Plant Breeding, Ecole Nationale D'Agriculture de Meknès, Meknès, Morocco; ^5^Department of Production, Protection and Biotechnology, Institut Agronomique et Vétérinaire Hassan II, Rabat, Morocco

**Keywords:** COVID-19, food behavior, food consumption, MENA region, North-Africa, Morocco

## Abstract

Alongside the dramatic impact on health systems, eating, shopping, and other food-related habits may have been affected by the COVID-19 crisis. This paper analyses the impacts of the COVID-19 pandemic on food shopping habits and food-related activities of a diverse sample of 340 adult consumers in Morocco. The study is based on an online survey conducted in Morocco from September 15 to November 5, 2020, utilizing a standardized questionnaire delivered in French and Arabic via Survey Monkey. The findings show that consumers' diet, shopping behavior, and food interactions have changed significantly. Indeed, the survey outcomes indicated (i) an increase in the consumption of local items owing to food safety concerns; (ii) an increase in online grocery shopping; (iii) a rise in panic buying and food hoarding; and (iv) an increase in culinary capabilities. The findings are expected to help guide Morocco's current emergency measures as well as long-term food-related policies.

## Introduction

The Coronavirus Disease 2019 (COVID-19), discovered in Wuhan (China) toward the end of 2019 (1), is now one of the most critical issues confronting humanity (2). Alongside the dramatic impact on health systems, the COVID-19 pandemic is expected to have dire effects on societies' socio-economic development and people's livelihoods worldwide (2). The pandemic is even considered a severe threat to achieving the Sustainable Development Goals (SDGs) encompassed in the 2030 Agenda for Sustainable Development (3).

Further, a growing body of studies indicates that COVID-19 has altered food systems (4, 5) with consequences on food and nutrition security (6–9). The COVID-19 epidemic created socio-economic shocks that impacted the global functioning of agricultural and food systems, as well as the food security situation of millions of people worldwide (10). Indeed, measures taken by governments to reduce and slow down the spread of the virus (e.g., lockdowns, mobility restrictions, shops closing) have affected several production sectors (e.g., agriculture) and value chains and disrupted international trade (11, 12). As a result, food production has generally declined during the pandemic, and all food chain stages have been disrupted (6, 13–17). Furthermore, survival psychology recognizes that individuals can experience behavioral adjustments due to specific circumstances such as natural disasters and health emergencies. These behavioral shifts may affect attitudes and behaviors related to food consumption (18). In this context, many articles show that the COVID-19 pandemic induced changes in food-related behaviors (19–22). Indeed, the pandemic has influenced food access and shopping behavior (23), food consumption habits and diets (24–28), as well as food wastage behavior (29). However, the COVID-19 pandemic effects are not alike across countries (11, 21, 27, 28, 30). El Bilali (31) argues that the pandemic is significantly affecting developing countries and vulnerable groups.

Morocco, a middle-income developing country, is the second most affected country by COVID-19 in Africa, after South Africa, and one of the most affected in the Near East and North Africa (NENA) region. As of February 21, 2021, Morocco recorded 480,948 confirmed cases of COVID-19 and 8,548 deaths (32). The first five confirmed cases of COVID-19 were reported in Morocco on March 2, 2020. Since then, the number of new cases per day has steadily increased to reach its peak of 6195 on November 13, 2020. Daily new cases have been slowly decreasing and reached 950 cases on December 29, 2020 (33). Morocco had initially successfully controlled the outbreak between March and May 2020 by taking several strong measures ranging from mobility restrictions (within the kingdom as well as travel bans) to calling for mandatory confinement. However, the government's priorities changed as the pandemic, and the related lockdown strategy, caused economic loss. As a consequence, Moroccan decision-makers eased precautionary measures on June 20, in favor of resuming economic activity and easing the financial constraints of individuals and businesses (34). Since then Moroccan government tries to have a balance between recovering the economy and saving lives by applying local measures instead of general ones, such as the lockdown applied in Casablanca during the first weeks of October 2020 (Table 1).

**Table 1:** Measures to contain the spread of the Coronavirus in Morocco.

**Date**	**Containment measure**
March 13 2020	Suspension of all passenger flights and ferry crossings to and from Algeria, Spain, and France
March 13 2020	Shutting down all schools and universities (from March 16)
March 15 2020	Suspension of all international flights, allowing only a minority of flights for foreigners wishing to leave the country Closure of mosques
March 19 2020	• Declaration of the state of health emergency from March 20 until April 20, 2020 • Closure of all non-essential shops and entertainment venues • Cancellation of all sport, cultural and art events • Suspension of intercity transportation
April 6 2020	Wearing face masks became mandatory starting from April 7
April 18 2020	Extension of the state of emergency until May 20 and later until June 10
June 9 2020	Announcement of a plan to ease the confinement measures and division of the country into two zones: Zone 1, where the health situation is under control; and Zone 2, where the situation is moderately controlled and most restrictions remain. Cafes, restaurants, cinemas and theaters remained closed in both zones.
July 9 2020	Resuming international flights, with access only for Moroccan citizens or for foreign residents in Morocco
September 4 2020	Allowing entrance of foreigners conditionally, through an invitation or a hotel reservation
October 5-14, 2020	Extended lockdown in Casablanca (the largest city of Morocco)
December 21 2020	Moroccan government announced imposing a 3-week curfew starting from December 23

*Le Monde (35); Maghreb Arabe Press (36); ONDA (37); Morocco World News (38); Hespress (39) and Medias24 (40)*.

These measures helped mitigate the public health threat, but many economic sectors, including the “informal sector,” were severely affected (34, 41). In this context, Haddad et al. (42) predict a decrease in Morocco's GDP and put that “*the main losses are concentrated in the regions that most contribute to the country's GDP, which coincide with the most densely populated areas, and that the most affected sectors are labor and flow-intensive*” (p. 10). Consequently, the government modified its priorities and eased the precautionary measures in June to re-launch the economy and reduce the financial pressure on citizens and enterprises (34). Also, Morocco created an emergency fund for the management of the COVID-19 pandemic (endowed initially with 10 billion MAD, about $1 billion) to upgrade health infrastructure and support the most affected economic sectors (43), including tourism, air transport, and some exporting sectors (e.g., textile and automotive sectors) (41).

The scholarly literature on the effects of the COVID-19 pandemic on food systems and consumption patterns has been so far mostly geographically biased; it focuses on Western and Southern Europe, North America, and China (30), while developing countries in general and those of the NENA region in particular, such as Morocco, have been overlooked. The analysis of the scholarly literature shows that most of the papers dealing with the COVID-19 emergency in Morocco focus on the dynamics of the spread of the virus as well as its health impacts (44–58). Other articles analyze the pandemic's socio-economic impacts in the kingdom (45, 59). Further papers address some specific impacts of the pandemic, such as on education (60, 61), transport (62), quality of life and wellbeing (63–65), and environmental pollution (66–68). Ouhsine et al. (69) analyze the impacts of the pandemic on solid waste generation at Moroccan households. However, the study was carried out only in two small municipalities in central Morocco (viz. Khenifra and Tighassaline) and does not specifically address food waste (it refers to a generic “organic fraction” without further distinction). Therefore, there is no comprehensive, nationwide analysis on food consumption behavior during the COVID-19 pandemic in Morocco. To fill this knowledge gap, the present article analyses the effects of the COVID-19 pandemic on food acquisition, access, and consumption in Morocco. In particular, the paper sheds light on how the COVID-19 emergency and the consequent confinement measures and lockdown, affected food-related behavior in Moroccan households.

## Data Collection and Methods

The study is based on an online survey conducted in Morocco using a standardized questionnaire^1^. It was conducted in Arabic and French using Survey Monkey from September 15 to November 5, 2020. The survey link was circulated by e-mail and social media (e.g., Facebook, LinkedIn, Instagram). The snowball-sampling approach was used in the research, and participants were requested to distribute the online questionnaire.

The study targets the general adult population (age >18 years) in Morocco. Participating in the survey was entirely optional, and there was no monetary incentive to do so. We used the non-probability sampling method. The study was performed in compliance with the principles set out in the Helsinki Declaration, and all procedures concerning research participants were authorized by the Western Michigan University Human Subjects Institutional Review Board (HSIRB). At the beginning of the survey, all participants were informed about the research's objective and context and gave their digital informed consent regarding privacy and information management policies.

The questionnaire consisted of 24 questions of different types (multiple-choice and one option), divided into three sections: [1] 9 questions on socio-demographics of the respondents (e.g. education level, gender, income, etc.); [2] 13 questions on food purchase and consumption behavior (e.g., food shopping behavior, food-related activities, food waste, etc.); and [3] 2 questions on positive and negative emotions during the pandemic. A pre-test was performed with 21 participants to assure data quality, and feedbacks were used to adjust the survey before its administration. The total of valid collected answers was 340.

For multiple-choice socio-demographic questions, response options depended on the question's nature. For example, for question 5: “How would you describe your household income compared to other households in Morocco?,” response options were: Much lower than most other households/ Slightly lower than most other households/About the same as most other households/Slightly higher than other households/Much higher than other households. For some multiple-choice questions, a Likert scale was used and response options were: never = 0; first time = 1; much less = 2; slightly less = 3; about the same = 4; moderately more = 5; much more = 6. For some other multiple-choice questions, response options were 5-point Likert scale: 1 (not at all), 2, 3, 4, 5 (very much).

The questionnaire was carefully constructed to reduce the threat of common method variance and mitigate the respondents' chance of misunderstanding the items. Also, a range of preventative measures was applied.

The survey findings were downloaded for analysis into SPSS (Statistical Package for Social Sciences) version 25.0. Descriptive statistics (means, standard deviations, percentages, frequencies) were calculated. The analysis of multiple responses was performed to draw the percentages of responses and cases as well as the trends. Since variables were nominal and ordinal, non-parametric tests were used. Furthermore, The Spearman correlation coefficient was calculated to evaluate the association between the respondents' variables and socio-demographic characteristics. Results were significant for *p* < 0.05.

## Results and Discussion

### Socio-Demographic Characteristics of the Study Participants

Table 2 describes the socio-demographic profiles of the survey participants. The findings indicate that 54.41% of the respondents are men, 41.76% are married with children, and 39.41% earn the same revenue as most other households in Morocco. In terms of occupation status, of all the interviewees, 63.82% were working (full-time or part-time), and 20.29% were students. In addition, most of the respondents were in middle age (46.18% of them were 25–45 years old). The sample was well-educated, with 76.18% of respondents holding a master's or a Ph.D. Furthermore, 13.24% of the cohort lost their employment or had their salary reduced because of COVID-19 (Table 2).

**Table 2:** Socio-demographic characteristics of the study participants (*n* = 340).

**Variable**		**Frequency**	**Percentage**	**Mean**	**Std. deviation**
		***N***	**%**		
Gender	Female	155	54.40	1.46	0.499
	Male	185	45.60		
Age	18–24	96	28.24	2.64	1.417
	25–34	77	22.65		
	35–44	80	23.53		
	45 and over	87	25.60		
Level of education	Secondary school and below	9	02.64	5.71	0.598
	University degree	72	21.18		
	Higher degree (MSc or Ph.D.)	259	76.18		
Income compared	Lower than most other Moroccan households	31	09.11	3.48	0.796
	About the same as most other Moroccan households	134	39.41		
	Higher than other Moroccan households	175	51.47		
Occupation	In paid work (full-time or part-time)	217	63.82	1.72	1.225
	Student	69	20.29		
	Unemployed and looking for work	19	05.59		
	Home duties or retired	35	10.29		
Household composition	Single person household	44	12.94	2.63	1.024
	Living with parents	107	31.47		
	Married with/without children	170	50.00		
	Extended family	16	04.71		
	Shared household, non-related	3	0.88		
Job loss/pay reduction	Yes	45	13.24	1.86	0.635
	No	295	86.76		

### Impact of COVID-19 on Food-Related Behaviors

As observed in several countries worldwide (72, 73), COVID-19 has transformed most participants' food shopping and procurement behavior in Morocco (Table 3). Firstly, 27.06% of the respondents indicated that they increased their purchase of local food products. Indeed, the consumption of local Moroccan food items has risen owing to food safety issues. As a consequence of the COVID-19 pandemic, concern over the transmission of the virus exists, and customers increasingly want to know where the food they purchase comes from. Consumers' irrational assumptions that foreign food items might pose a safety danger involved a preference for local food products. Also, 20.59% of the respondents stated that they ordered more groceries online. Meanwhile, 17.35% of respondents indicated that they ordered more food online from a full-service or fast-food restaurant or by a delivery application (Table 3).

**Table 3:** Behavioral change during the COVID-19 pandemic (*n* = 340).

Category^*^	**Never**		**First time**		**Much Less**		**Slightly less**		**About the same**		**Moderately more**		**Much more**		**Mean**	**Std. Deviation**
	**Frequency**	**Percentage**	**Frequency**	**Percentage**	**Frequency**	**Percentage**	**Frequency**	**Percentage**	**Frequency**	**Percentage**	**Frequency**	**Percentage**	**Frequency**	**Percentage**		
Buying local food	5	1.5	3	0.9	0	0	18	5.3	222	65.1	60	17.65	32	9.41	4.33	0.843
Ordering groceries online	213	62.5	16	4.7	6	1.76	6	1.76	36	10.8	34	10	29	8.53	5.96	1.665
Buying food in person from a large supermarket	24	7.0	5	1.5	50	14.7	60	17.65	125	36.7	44	13	32	9.41	4.01	1.430
Buying food in person from a small supermarket or grocery store	8	2.3	5	1.5	30	8.82	55	16.18	140	41.1	60	17.65	42	12.35	4.11	1.233
Meals delivered from a restaurant or by a delivery application	177	51.9	8	2.3	27	7.94	19	5.6	50	14.7	40	11.76	19	5.6	5.51	1.831

* *Scale values: never = 0; first time = 1; much less = 2; slightly less = 3; about the same = 4; moderately more = 5; much more = 6*.

Moreover, since shopping in a supermarket has a perceived risk, participants buying patterns have changed. On the one hand, more respondents purchase their groceries online to escape crowded shops, thereby accelerating food retailers' digitization (74). Responding to this growing demand, some Moroccan hypermarkets increased their delivery capacity and launched their e-commerce platforms for the first time. Marjane, the Moroccan hypermarket, launched a delivery App and partnered with the Spanish distribution platform Glovo (75). Also, in April 2020, Carrefour launched, in partnership with Jumia Food, a free home delivery service in major Moroccan cities (76). However, these channels did not grow as high as may have been the case in other countries. Several significant hurdles limit online shopping development in Morocco, such as the lack of online payment systems and low Internet penetration (77).

Secondly, 52.65% of the participants said they had stocked up on food since COVID-19 became severe. Indeed, just before the confinement in March 2020, a rush toward supermarkets has been observed in Morocco, and demand for flour and grains has jumped. Moroccans were panicking over the Coronavirus and stocking up in droves. Hence a surge in food prices. Despite promises from the government and stores that the food supply system can satisfy the extraordinary hoarding caused by the epidemic, pasta, wheat, and salt shelves have been depleted (78).

Thirdly, 54.71% of the participants specified that they go shopping less than usual, and 35.29% indicated that they buy more than usual on each trip to the grocery store (Table 4). Indeed, since shopping in person in a supermarket has a perceived risk and induces fears of being close to others, participants' buying patterns have changed. Despite many protective measures and regulations applied by supermarkets (e.g., the installation of protective barriers, frequent cleaning, provision of masks, gloves, and disinfectants, etc.), for many consumers, shopping at a grocery store poses an evident danger (79, 80). As observed in several countries, most study participants reduced the number of shopping trips and were shopping less than usual, buying more on each trip to diminish store visits and limiting their perceived risk of exposure to COVID-19 (Table 4).

**Table 4:** Shopping behavior and purchasing changes during the COVID-19 pandemic (*n* = 340).

**Variable**	**Statement**	**Frequency**	**Percentage**
Shopping behavior change	I go shopping less than usual	186	54.71
	I go shopping like I used to	102	30
	I go shopping more than usual	52	15.29
Change in food purchase	I buy more than usual	120	35.29
	I buy as same as usual	165	48.53
	I buy less than usual	55	16.17

The Spearman correlation test results revealed that age significantly affected some behaviors and habits (Table 5). For example, age had a very significant effect (*p* < 0.05) on the number of shopping trips. Aware that the risk for severe illness with COVID-19 increases with age, older participants go shopping less than usual, buying more on each trip to limit their perceived risk of exposure to COVID-19. Also, old participants, concerned for their families and the long-term outlook, stocked more food than younger ones (Table 5). Panic buying and stockpiling were shaped by several factors, including socio-demographic factors (e.g., culture, income, gender, and personality). Household preferences and attitudes and product categories may also be differentially affected over time (81). Overall, individuals in different age groups have responded differently to the health crisis (82).

**Table 5:** Socio-demographic effects on food shopping behavior.

	**Gender**	**Age**	**Level of education**	**Household income**	**Profession**	**Family composition**	**Job loss or pay reduction**
Shopping behavior change	0.234	0.000^*^	1.33	0.420	0.818	0.19	0.554
Change in food purchase	0.851	0.379	0.470	0.175	0.852	0.574	0.27
Stocking up food behavior	0.931	0.018^*^	0.866	0.328	0.454	0.191	0.825

* *Significant at p < 0.05*.

Also, the results highlight some changes in food-related activities. Indeed, 38.53% of participants reported eating more with family members, 63.53% were cooking and making food even more often, and 59.12% spent a lot of time cooking. Furthermore, 27.36% made less easy meals (e.g., instant foods, frozen foods, etc.). Additionally, with the closure of the HORECA channel (hotels, restaurants, and catering), consumers have moved from out-of-home to home-based eating, with more cooking and baking at home. Trying to recreate the restaurant experience, many consumers rediscovered home cooking. Indeed, it is much easier to find the time for these activities and try new recipes with the confinement (83–85). Moreover, with restaurants, coffee shops, and cultural institutions closed, entertainment options became restricted, and eating with family and cooking became new entertaining activities.

## Conclusion

The health and economic crisis triggered by the COVID-19 pandemic has had disorderly societal, economic, and psychological effects on food behaviors and consumption patterns, contributing to an impending global food emergency. However, impacts differ from country to country, and national data is essential for research and comprehension. In this context, this study examines the effects of the COVID-19 pandemic on food behavior in Morocco based on a cross-sectional survey involving 340 participants. The survey results show that the COVID-19 pandemic, and preventive actions taken by the Moroccan government, had affected food-related behaviors and habits. Undoubtedly, there have been apparent modifications in the way participants are shopping and interacting with food. To our knowledge, this is the first study about the perceptions of the impacts of COVID-19 on food behaviors in Morocco. Given that the COVID-19 pandemic is new and uncertain how long it will last, data and knowledge are needed to assess its effect on food consumption patterns. In addition, since there is no widespread literature on contemporary pandemics outside of SARS, the COVID-19 study will guide comprehension and even predict the potential of shock and crisis research (18). This and other future research will serve as a foundation for organizational and government readiness for future shocks and pandemic outbreaks.

The sample bias is the main limitation of this study. The survey participants were chosen at random and recruited voluntarily. As a self-administered questionnaire, it was performed by volunteers who were not compensated. Therefore, only persons driven by an interest in the topic participated in the survey (cf. self-selection of the sample). Consequently, our sample does not reflect the general population in Morocco. For example, high-educated individuals were overrepresented in our sample. In general, low-educated individuals are frequently underrepresented in surveys (86). Furthermore, online surveys tend to exclude those who are web-illiterate as well as the elderly. More specifically, in the NENA region, poor households and informal workers are the least likely to be heard through online surveys. Inadequate infrastructure, low computer literacy, and lack of money to purchase a device or internet subscription can limit their access to the Internet, resulting in less participation in online surveys (87). The limitations mentioned above are frequent in Computer Assisted Web Interviewing (CAWI), which is now usually applied in surveys (88–90). This bias limits the ability to generalize survey findings to the whole Moroccan population. However, because of the COVID-19 epidemic, online surveys can collect data from a distance, which is a distinct benefit when social distancing is necessary and face-to-face research is problematic.

So far, the scholarly literature on the impacts of the COVID-19 pandemic on food systems and consumer behavior has been geographically biased, focusing on Western and Southern Europe, North America, and China (30). In contrast, developing countries in general, and those of the NENA region in particular, such as Morocco, have been overlooked. To the best of our knowledge, this is the first study in Morocco on consumers' perceptions of the effects of COVID-19 on food behaviors. Given that the COVID-19 pandemic is new and its duration is unknown, data and knowledge are required to assess its impact on food consumption patterns. The findings of the paper confirm that the final results of COVID-19 will most likely differ from country to country, depending not only on the epidemiological situation but also, among other factors, on the baseline situation and shock resilience (91). As highlighted by El Bilali (31) “The pandemic immediate impacts vary from a country to another depending, inter alia, on the epidemiological situation, lockdown and confinement measures, pre-COVID socio-economic development level” (p. 59). This and other future researches will serve as a foundation for organizational and government readiness for future shocks and pandemic occurrences. The study's findings are also helpful for developing evidence-based policy in Morocco and the NENA area as a whole during the post-pandemic recovery period.

Finally, many researchers questioned if these changes in consumers' behaviors and diets are permanent or transient. However, since the COVID-19 infection is new and still unfolding, and the channels of influence are multiple and interconnected globally, the precise consequences in the future on food habits are unknown. Further, the pandemic is far from over, and some countries still face significant epidemics, but even those who currently control the virus fear upcoming waves, especially with the spread of more contagious variants (32). The possibility of new infections and waves could result in new lockdowns or continuity of the current tight measures over the coming months, contributing to more disruption of economic activity and food-related activities.

## Data Availability Statement

The raw data supporting the conclusions of this article will be made available by the authors, without undue reservation.

## Ethics Statement

The studies involving human participants were reviewed and approved by the Western Michigan University Human Subjects Institutional Review Board (HSIRB). The patients/participants provided their written informed consent to participate in this study.

## Author Contributions

TB and HE: conceptualization, writing—original draft preparation, and project administration. TB, HE, and CB: methodology and formal analysis. CB: software, validation, and data curation. AA and SA: investigation. TB, HE, CB, AA, and SA: writing—review and editing. TB: funding acquisition. All authors have read and agreed to the published version of the manuscript.

## Funding

The publication of this article was funded by the Qatar National Library.

## Conflict of Interest

The authors declare that the research was conducted in the absence of any commercial or financial relationships that could be construed as a potential conflict of interest.

## Publisher's Note

All claims expressed in this article are solely those of the authors and do not necessarily represent those of their affiliated organizations, or those of the publisher, the editors and the reviewers. Any product that may be evaluated in this article, or claim that may be made by its manufacturer, is not guaranteed or endorsed by the publisher.

## Footnotes

^1^This survey was part of the “Consumer Agency, Food Consumption Behavior, and the Novel Coronavirus (COVID-19) Outbreak” international research project promoted by the Food Industry Research and Education Center (FIRE) at the Western Michigan University. The international research included teams from China, Germany, Netherlands, Qatar, Turkey, UK, and the USA (70). The questionnaire used in the online survey was informed by the Food Consumption Changes 2020 survey of the West Michigan University (70) and the COVID-19 Survey of the United Nations System Standing Committee on Nutrition (UNSCN) (71).
